# Identification of Multiple HPV Types on Spermatozoa from Human Sperm Donors

**DOI:** 10.1371/journal.pone.0018095

**Published:** 2011-03-29

**Authors:** Maja D. Kaspersen, Peter B. Larsen, Hans Jakob Ingerslev, Jens Fedder, Gert Bruun Petersen, Jesper Bonde, Per Höllsberg

**Affiliations:** 1 Department of Medical Microbiology and Immunology, Aarhus University, Aarhus, Denmark; 2 Cryos International Sperm Bank, Aarhus, Denmark; 3 The Fertility Clinic, Aarhus University Hospital, Aarhus, Denmark; 4 Laboratory of Reproductive Biology, Scientific Unit and Fertility Clinic, The Regional Hospital Horsens, Horsens, Denmark; 5 Pathology Division, Clinical Research Center, Hvidovre Hospital, Hvidovre, Denmark; Institut National de la Santé et de la Recherche Médicale, France

## Abstract

Human papillomaviruses (HPV) may cause sexually transmitted disease. High-risk types of HPV are involved in the development of cervical cell dysplasia, whereas low-risk types may cause genital condyloma. Despite the association between HPV and cancer, donor sperm need not be tested for HPV according to European regulations. Consequently, the potential health risk of HPV transmission by donor bank sperm has not been elucidated, nor is it known how HPV is associated with sperm. The presence of 35 types of HPV was examined on DNA from semen samples of 188 Danish sperm donors using a sensitive HPV array. To examine whether HPV was associated with the sperm, in situ hybridization were performed with HPV-6, HPV-16 and -18, and HPV-31-specific probes. The prevalence of HPV-positive sperm donors was 16.0% and in 66.7% of these individuals high-risk types of HPV were detected. In 5.3% of sperm donors, two or more HPV types were detected. Among all identified HPV types, 61.9% were high-risk types. In situ hybridization experiments identified HPV genomes particularly protruding from the equatorial segment and the tail of the sperm. Semen samples from more than one in seven healthy Danish donors contain HPV, most of them of high-risk types binding to the equatorial segment of the sperm cell. Most HPV-positive sperm showed decreased staining with DAPI, indicative of reduced content of DNA. Our data demonstrate that oncogenic HPV types are frequent in men.

## Introduction

The family of human papillomavirus (HPV) encompasses more than 100 types, of which 40 have been isolated from the genital tract [1]. A number of these (HPV-16, -18, -26, -31, -33, -35, -39, -45, -51, -52, -53, -56, -58, -59, -66, -68, -70, -73, -82, and -85) are grouped as high-risk types, because they are involved in the development of cervical neoplasia in persistently infected women [2]. Of these types, only HPV-16 and -18 are included in the HPV vaccines. Thus, HPV remains a risk factor for cervical neoplasia in vaccinated women. In addition, a number of low-risk types are also sexually transmitted and may cause genitial condyloma [3].

Despite the clinical importance of HPV in lower genital tract cancers, little attention has been given to the transmission of HPV through semen. Several studies have identified HPV in human semen, mainly from men recruited at fertility or maternity clinics, with a prevalence ranging from 4.5–64.3% [4], [5], [6], [7], [8], [9], [10], [11], [12], [13]. The variability reflects differences in clinical characteristics and limitations in both the number of analyzed study subjects and HPV types. Whether or not the presence of HPV in semen has consequences for sperm functionality has yet to be resolved. Conflicting conclusions have been drawn regarding sperm concentration, motility, and vitality as well as semen volume and pH [4], [5], [10], [14], [15], [16], [17], [18], [19].

HPV may potentially enter the uterus transmitted by sperm. Attempts to wash off HPV DNA from sperm have failed [9], [12], [16], suggesting either a very strong binding of HPV to a receptor on the sperm cell surface or an entrance of HPV into the sperm cell. Indeed, sperm-associated HPV DNA fragments have been isolated from mouse blastocysts and cells of the murine endometrium [20], and HPV E6 mRNA sequences has been detected in sperm cell specimens [21]. Whether or not human sperm express a specific receptor for HPV is not known, but Pérez-Andino et al. [22] demonstrated recently that HPV-16 capsids adsorb to distinct sites on the sperm head surface at the equatorial segment, suggesting that these sites may mediate HPV binding.

The use of semen from sperm banks for insemination highlights the importance of knowing the risk of attracting HPV infections during these procedures. The prevalence by which donor semen contains HPV high-risk types not covered by the current HPV vaccines becomes important, since semen samples are not required to be tested for the presence of HPV. We therefore examined the prevalence and types of HPV in 188 semen donors using a sensitive array technology. Finally, we provide evidence for in vivo association of HPV with a specific region on the sperm head surface. Noticeably, HPV-positive sperm had decreased amount of DNA.

## Materials and Methods

### Ethics statement

The study was conducted according to the Helsinki declaration and the sperm donors had signed a declaration of consent stating that the donated semen may be used for scientific intentions. Samples were anonymized and no information is referable to any individual. The Ethical Committee of Science for the Region of Middle Jutland has approved the study (M-20100238).

### Donors and samples

A total of 267 semen samples from 188 sperm donors were obtained from three Danish units of Cryos International Sperm Bank. From 34 of the donors, multiple ejaculates were examined: two samples were examined from 21 donors, three samples from 5 donors, four samples from 2 donors, seven samples from one donor, eight samples from 4 donors, and ten samples from 1 donor. Seventy-four (39.4%) of these samples were from first time donors not yet approved for sperm donation and therefore unselected and representing the general male population, whereas the remaining 114 samples came from approved active sperm donors. The only exclusion criterion for the first-time donors was no sperm. Approved donors had been examined and excluded if they had a predisposition to certain inheritable diseases (http://dk.cryosinternational.com/clinics/screening.aspx), previous known genital infections, current infection of *C. trachomatis* or *N. gonorrhoae* (swab), or sero-positivity towards human immunodeficiency virus type1 and 2, human T-cell lymphotropic virus type 1 and 2, hepatitis B and C, and *T. pallidum*. The age range of the donors at the time of sperm donation was 18 to 48 years, with a mean age of 25 years (SD = 6.1). Donors are not selected based on ethnicity and therefore probably reflect that of the general Danish population of young men.

### Sperm sample handling and DNA extraction

Semen samples were visually analyzed, frozen in liquid nitrogen either untreated or after addition of 0.33 mL SpermCryos Allround (SCA) freeze medium (ProVitro, Odense, Denmark) per 1 mL semen, and stored at −196°C until further analysis. DNA was extracted using a Qiagen DNA extraction kit (Qiagen, Copenhagen, Denmark) and the robotic QiaCube (Qiagen) according to the manufacturer's instructions.

### Array-based detection of type-specific HPV in semen

HPV type-specific DNA fragments of 380–450 base pairs were amplified using type specific primers for the conserved L1 region. During amplification, the products were labelled with biotin. HPV-positivity of the samples was determined using the CLART HPV 2 Clinical Array (Genomica, Madrid, Spain) [23]. This array is based on the identification of biotinylated PCR products on a 2×2 mm glass platform, which is divided into 120 immobilized cDNA spots, coated with probes specific for one of 35 HPV types in non-grouped triplicates. The biotinylated products hybridize to their specific probes, and the biotin on the amplified and immobilized probes are recognized by a peroxidase-conjugated streptavidin. The array design includes a control for the presence of genomic DNA consisting of an 892 base pair PCR amplification of the humane cystic fibrosis transmembrane conductance regulator (CFTR) gene, and an amplification control consisting of plasmid CFTR of 1202 base pairs. The CLART HPV 2 Clinical Array detects with high sensitivity low risk HPV types 6, 11, 40, 42, 43, 44, 54, 61, 62, 71, 72, 81, 83, 84, 89, and high-risk HPV types 16, 18, 26, 31, 33, 35, 39, 45, 51, 52, 53, 56, 58, 59, 66, 68, 70, 73, 82, and 85. The procedure involves an initial denaturation of DNA by incubation at 95°C for 10 min followed by an immediate cooling on ice. The denatured products were diluted 1∶20 in Hybridization Solution Buffer, and 100 µl was added to each prewashed array well. The probes were allowed to hybridize at 60°C on a thermomixer for 1 hour. The wells were washed twice with wash buffer and then incubated with a simultaneously blocking and conjugating buffer at 30°C for 15 min on a shaking thermomixer. Subsequently, the wells were washed three times before addition of 100 µl Developer per well. The developing process was carried out without agitation at 25°C for 10 min, and immediately read by a Clinical Arrays Reader. The CLART HPV 2 Clinical Array has been developed for diagnostic use. According to the product information, this array performs with a diagnostic sensitivity of 100% for 29 of the HPV types, >91% for 5 types, and with undetermined specificity for type 85. The Genomica CLART assay was chosen because of the great robustness and reliability of this assay. The Clinical Research Center, Pathology Division, Hvidovre Hospital, participated in a recent quality assurance panel distributed by the WHO Lab Net Work. The results demonstrated that the CLART assay in our hands repeatedly detected from 25 copies HPV16 in 5 µl H_2_O to >5000 copies in 5 µl H_2_O.

### In situ hybridization

HPV type 16 and 6 were visualized in sperm by in situ hybridization using a ZytoFast HPV-Typing Kit (ZytoVision, Bremerhaven, Germany). The in situ hybridization procedure was carried out according to the manufacture's instructions. Briefly, 10 µl raw semen were smeared and allowed to air dry on a Thermo Scientific Superfrost Plus object glass (Thermo Scientific, Copenhagen, Denmark) and fixed in 4% (v/v) formalin. The cells were treated with a Pepsin Solution for 5 min at 37°C, and the proteolytic reaction was stopped in 100% ethanol, after which the cells were rinsed in deionized water. Hybridization of biotinylated HPV type 16 and 6 specific DNA probes was carried out with an initial denaturation for 10 min at 75°C, and hybridization for 1 hour at 37°C. Unhybridized probes were removed by washing in TBS and distilled water, and AP-anti-Biotin and AP-substrate were consecutively applied, each with a 30 min incubation at 37°C. Positive and negative controls for HPV-16 detection were included in the form of sections of tonsil biopsies. Cover slips were mounted with a mix of polyvinyl alcohol and glycerol, and the microscope slides were analyzed on a Leica LEITZ DM microscope with a 100× magnification.

### Fluorescence in situ hybridization

The HPV types 6, 16, 18, and 31 were visualized in sperm by fluorescence in situ hybridization (FISH) using the above-mentioned ZytoFast HPV-Typing Kit (ZytoVision, Bremerhaven, Germany) and a PE-conjugated streptavidin (BD Biosciences Pharmingen, San Diego, CA). The FISH procedure was carried out according to the instructions of the ZytoFast HPV-Typing Kit with few modifications. Briefly, 20 µl raw semen were smeared and allowed to air dry on a Thermo Scientific Superfrost Plus object glass (Thermo Scientific, Copenhagen, Denmark) and fixed in 4% (v/v) formalin. The cells were treated with a Pepsin Solution for 5 min at 37°C, and the proteolytic reaction was stopped in 100% ethanol, after which the cells were rinsed in deionized water. In order to avoid unspecific binding of streptavidin endogenous biotin were blocked with the AP-anti-Biotin from the ZytoFast HPV-Typing Kit. Blocking was carried out for 30 min at 37°C. Hybridization of biotinylated HPV type 6/11, 16/18, and 31/33 specific DNA probes was carried out with an initial denaturation for 10 min at 75°C, and hybridization for 1 hour at 37°C. Unhybridized probes were removed by washing in TBS and distilled water, and PE-conjugated streptavidin was applied with a 30 min incubation at 37°C, followed by a thoroughly washing. Finally, 4′,6-diamidino-2-phenylindole dihydrochloride (DAPI) (300 µM) (Sigma-Aldrich, Brøndby, Denmark) was applied for 5 min without subsequent washing. Cover slips were mounted with a mix of polyvinyl alcohol and glycerol, and the microscope slides were analyzed on a Zeiss confocal microscope with a 63× magnification. The specificity of the fluorescent signal was verified by including preparations from three HPV-negative (as determined with the HPV array) semen samples probed with a mix of all three HPV probes and by checking for convergence of signaling through channels capturing wavelengths different from those excited by PE.

## Results

**Figure 1:**
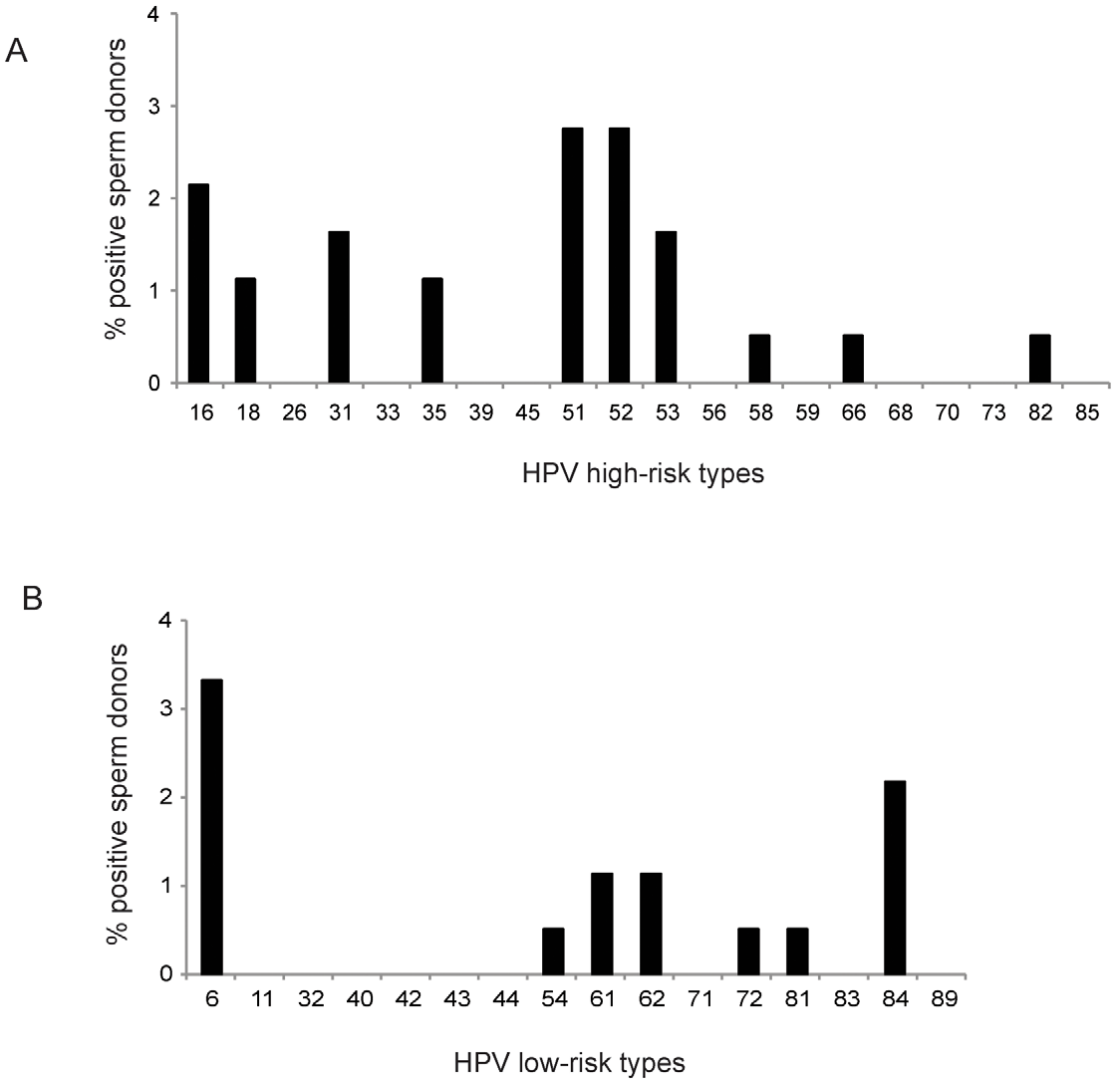
HPV frequency and type distribution in Danish donor semen, analyzed using a sensitive HPV type-specific array. (**A**): Frequency of twenty high-risk types, given as percent of all tested donors. (**B**): Frequency of fifteen low-risk types, given as percent of all tested donors.

Semen samples from 188 healthy Danish sperm donors were analyzed for the presence of 20 high-risk and 15 low-risk types of HPV (Figures 1A and 1B). From 10.6% (20/188) of the donors high-risk HPV types could be detected, whereas low-risk types were detected in 8.0% (15/188) of donors. Among the high-risk types, HPV-51 and -52 were most frequently detected (each 2.7% (5/188)), whereas HPV-16 was found in 2.1% (4/188) of the donors (Figure 1A). Among low-risk types, HPV-6 was detected in 3.2% (6/188) and HPV-84 in 2.1% (4/188) of donors (Figure 1B). Taken together, 16.0% (30/188) of sperm donors were positive for the tested HPV types and 10 of the donors (5.3%) had double or triple HPV infections (8 double and 2 triple). Semen donor positive for HPV was in 33.3% (10/30) of the cases infected with additional HPV types. Among all the detected HPV types, 61.9% (26/42) were high-risk types and 38.1% (16/42) were low-risk types.

Despite donor acceptance criteria such as restricted sexual behavior and no current or previous signs of genital HPV infection, no significant difference in positivity was observed between selected and unselected donors (15.8% (18/114) versus 16.2% (12/74), respectively).

**Figure 2:**
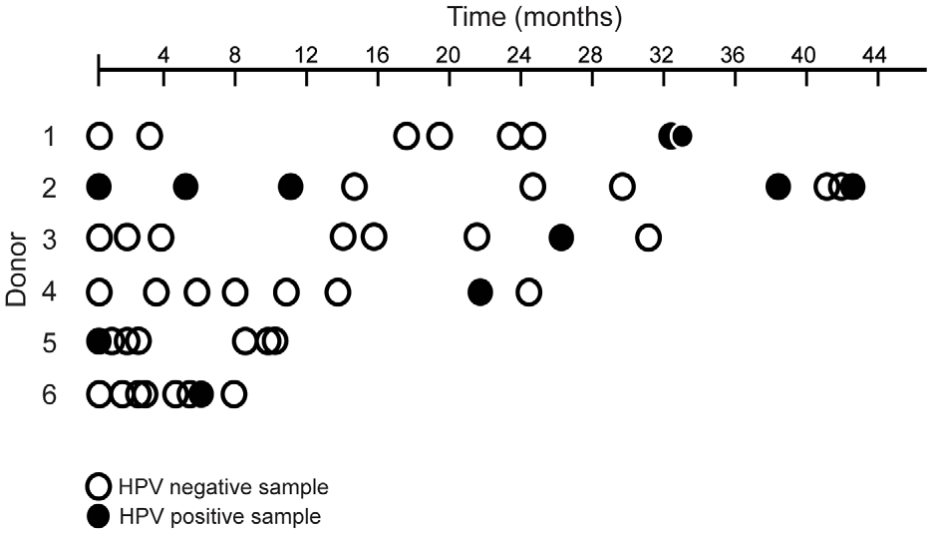
Longitudinal analysis of HPV-positive sperm samples. HPV is not continuously shed in semen. Circles on a relative time scale image series of ejaculates from 6 different donors, occasionally positive for an HPV. Filled circles represent HPV positive samples; open circles represent HPV negative samples. Starting points are given by the first analyzed ejaculate for each donor. Series of ejaculates from six different donors were found positive for HPV type 84; type 53, 31, and 52; type 62; type 61; type 54; and type 52, respectively.

From the 188 donors, a total of 267 ejaculates were analyzed for presence of 35 types of HPV. Series of 8–10 ejaculates from 6 different donors delivered over various time ranges revealed an inconsistent pattern of viral shedding in the semen (Figure 2).

**Figure 3:**
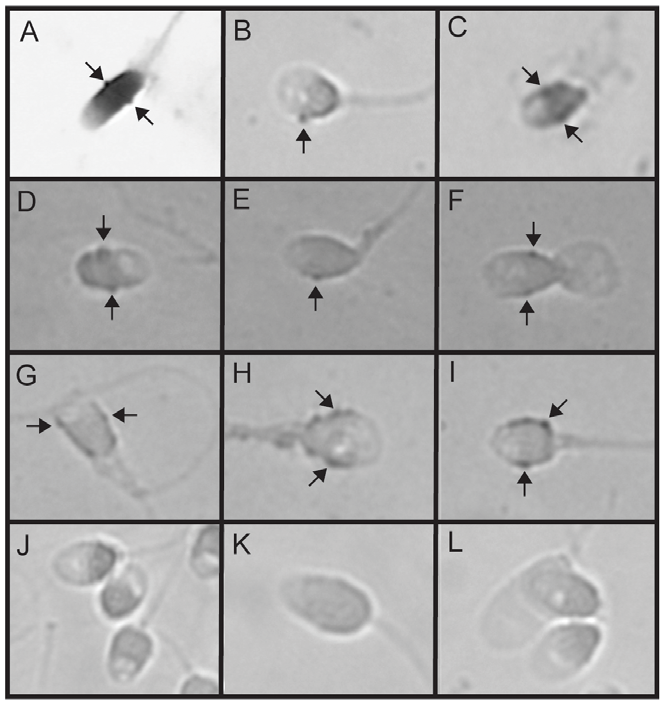
In situ hybridization on human sperm using HPV type-specific probes. Specific hybridizations are indicated by arrows. (**A**–**C**): In situ hybridization on an HPV-16 positive sperm sample using a probe specific for HPV-16. The sample in picture **A** was simultaneously background stained with hematoxylin. (**D**–**I**): In situ hybridization on an HPV-6 positive sperm sample using a probe specific for HPV-6. (**J**–**L**): In situ hybridization on an HPV negative sperm sample using a probe specific HPV-6. This figure is a representative of three independent in situ hybridization experiments using two different HPV-6 positive samples and two different HPV-16 positive samples.

**Figure 4:**
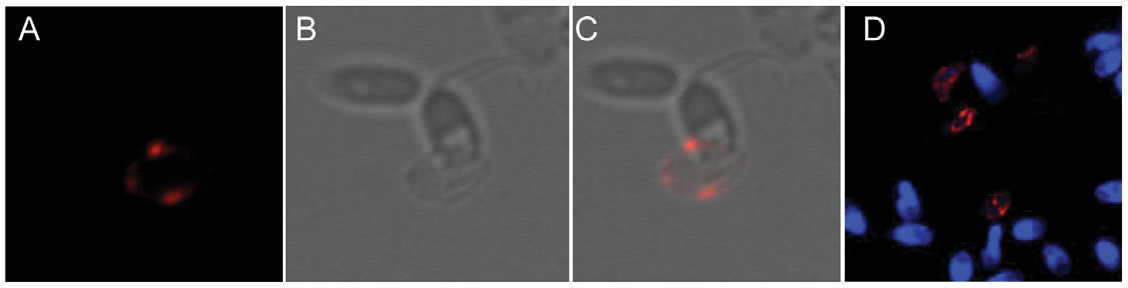
Binding pattern of HPV to sperm using fluorescence in situ hybridization with HPV type-specific probes. (**A–C**): Close-up picture of sperm of which one is positive for HPV type 18. Picture B shows the sperm present in picture A, captured by differential interference contrast (DIC). Picture C shows an overlay of A and B. (**D**): Close-up picture of HPV-negative and HPV-18 positive sperm cell from an HPV-18-positive semen sample stained for HPV (red) and DNA (blue). Note the lack of DNA staining characteristic of the majority of HPV positive sperm. Lack of or decreased DNA staining was seen in all HPV-positive sperm.

To address whether in vivo association between HPV sperm was present, we took advantage of samples already characterized for the presence of HPV. Sperm samples positive for HPV-6, HPV-16, HPV-18, or HPV-31 were hybridized with a specific probe against the respective type, and the HPV-probe-sandwiches were visualized (Figures 3A–3I and 4). This revealed characteristic protrusions at or near the equatorial segment of the sperm head (Figures 3A–C). Likewise, when sperm from HPV-6, -18, or -31 positive donors were hybridized with HPV-6, -18, or 31-specific probes, similar protrusions were identified (Figures 3D–I and 4). However, when sperm from an HPV negative donor were hybridized with an HPV specific probe, there was no specific binding (Figures 3J–L and 4). HPV-infected tonsil biopsies were included as positive controls (data not shown). These data indicate that HPV-6, -16, -18, and -31 bind to the sperm cell head particularly at or near the equatorial segment in vivo.

Interestingly, fluorescence in situ hybridization results revealed decreased DAPI staining of all sperm binding HPV, the majority with no detectable DAPI stain (Figures 4C and D).

**Figure 5:**
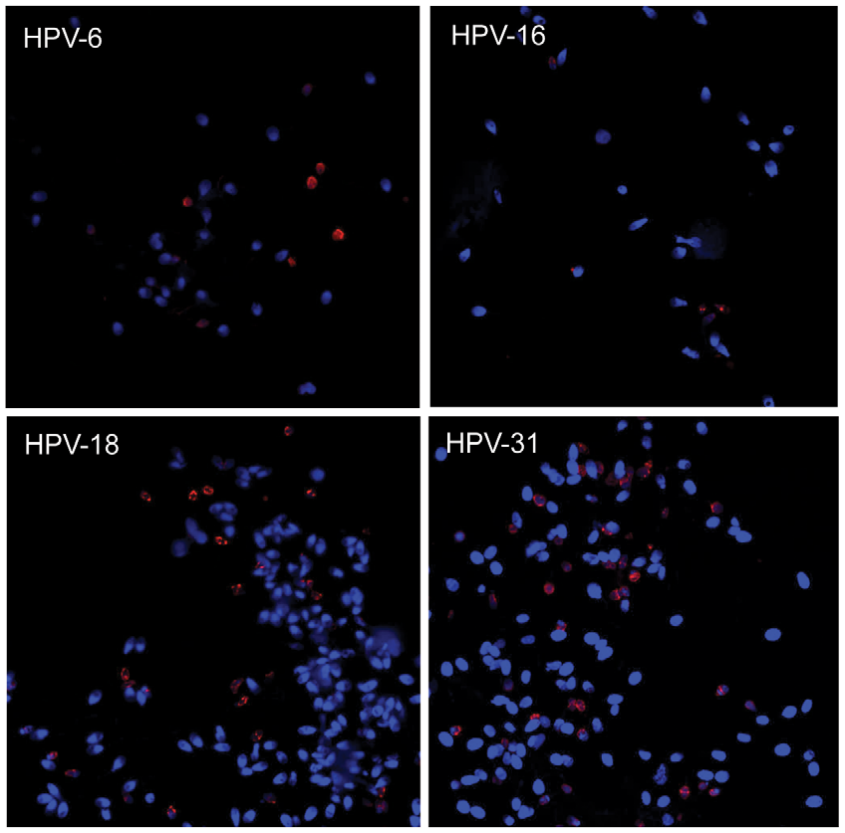
Frequency of HPV-positive sperm using fluorescence in situ hybridization with HPV type-specific probes. Low magnification pictures illustrating the quantity of HPV in positive semen samples. Samples are positive for HPV-6, -16, -18, and -31, respectively. Samples are stained for HPV with PE-conjugated streptavidin (red) and stained for DNA with DAPI (blue). Pictures are representatives of coincidental spots of the preparation slides, which are representatives of HPV positive semen samples from 9 donors (HPV-6, -16, -18, and -31 represented by 2–3 donors each).

Three fourths of samples, which could be semi-quantified by FISH and the available probes, were chosen for quantification by manual counting. In Figure 5, the frequency of HPV-positive sperm in ejaculates from three donors is illustrated. A total of 2196 sperm were counted from 9 HPV-positive donors giving an overall mean of HPV-positive sperm of 7.4% and a median of 5%. The range was 1–16.5%.

## Discussion

Despite the likely pathogenic importance of oncogenic HPV types in semen, the frequency of high-risk and low-risk HPV types in healthy semen donors has not received much attention. A very recent paper on 60 semen samples from healthy individuals found that 3.3% contained HPV [24]. We used a sensitive HPV array to detect 35 types, encompassing 20 high-risk and 15 low-risk types of HPV. The HPV types included in this array constitute the most common ano-genital types. We cannot exclude, however, that our HPV-negative semen samples might contain HPV types different from the ones recognized by the array, but the majority of the non-included HPV types gives cutaneous infections and are therefore not likely to be found in semen or persist inside genitals. We found that 16% of healthy semen donors harbor HPV in their semen and 11% of semen samples harbor high-risk types.

On average, we found that HPV-positive samples contained 7.4% HPV-positive sperm. However, this number varied considerably from 1–16.5% and moreover, we do not know how much HPV DNA is needed to give a positive signal in our fluorescence in situ hybridization analysis. We have not determined the viral load in the samples, since no international consensus as to the relevance of viral load for HPV infection has been agreed upon, and to our knowledge no qPCR has been clinically validated. Thus, the significance of HPV transmission from HPV-positive semen versus direct mucosal contact remains to be determined. Anyhow, semen cannot be excluded as a possible risk factor for HPV transmission and testing donor semen for HPV should be considered.

The finding of HPV in 16% of semen samples is comparable with a recent paper on HPV frequency in Danish women [25]. In this study, an overall HPV prevalence of 26% was found with a peak incidence of 50% in women between 20 and 24 years of age. Among all age groups, high-risk HPV types were found in 19% of women with normal cytology.

The prevalence of HPV types may vary with the geographical locations. In men from Africa, less than 55% of the high-risk types were among HPV types 16, 18, 31, 33, 45, 52, and 58 [5]. The most prevalent high-risk types in our analysis were HPV types 51, 52, 16, 31, and 53, whereas e.g. HPV-33 and HPV-45 were not detected among our samples. This suggests that investigations as presented here should be performed at different geographical locations to get the most adequate picture of the distribution of HPV types in semen. This is of direct importance for the development of vaccines, which may need to be designed more specifically for individual countries.

Using fluorescence in situ hybridization to visualize viral particles concomitantly with sperm DNA, we noticed a significant decrease in sperm DNA staining by DAPI, when HPV was associated. The mechanism for this is unknown, but may be associated with an HPV-induced apoptosis. Other reports, however, have stated that HPV is still detectable in sperm samples after sperm wash [9], [12], [16] suggesting the possibility of viral transfer to the cervix and the intrauterine cavity via sperm post intercourse or during intrauterine insemination (IUI). Regardless of the viability of the sperm, a transmission of the virus from the sperm surface to the female cervical epithelia could imaginably occur through simple contact between the sperm cell and the epithelia cell. HPV have been isolated from endometrial carcinomas [26], [27], but whether HPV is the causative agent of these carcinomas remains to be defined. Moreover, an association between HPV infections and decreased pregnancy rate has been reported [28].

Considering the general infertility population, assisted reproductive technology procedures, like intracytoplasmic sperm injection, involve no natural selection of the sperm cell, which means that these procedures have a plausible risk of injecting sperm containing HPV. Our observation of HPV-6, -16, -18, and -31 association to or near the sperm head equatorial segment is consistent with the finding of Pérez-Andino et al. [22], who recently showed that pseudovirions of HPV 16 capsid proteins adsorb to this exact same location on the sperm cell. Thus a specific receptor for HPV may be located at or near the equatorial segment.

A proportion of women immunized with the currently available HPV vaccines may still attract cervical cancer. Besides the role of HPV in dysplasia of cervix, dysplasia of genital tissues also occurs in males. This indicates that it may be important to further characterize the circulating HPV high-risk types in both women and men. Our data have contributed to increased knowledge of the distribution pattern of HPV in healthy men. This information may be useful for improving HPV vaccine coverage.
